# Controlling pore-scale processes to tame subsurface biomineralization

**DOI:** 10.1007/s11157-021-09603-y

**Published:** 2022-01-21

**Authors:** Joaquin Jimenez-Martinez, Jen Nguyen, Dani Or

**Affiliations:** 1 Department of Water Resources and Drinking Water, Eawag, Dübendorf, Switzerland; 2 Department of Civil, Environmental and Geomatic Engineering, ETH Zurich, Zürich, Switzerland; 3 Department of Microbiology and Immunology, University of British Columbia, Vancouver, BC V6T 1Z3 Canada; 4 School of Biomedical Engineering, University of British Columbia, Vancouver, BC V6T 1Z3 Canada; 5 Division of Hydrologic Sciences, Desert Research Institute, Reno, NV USA

**Keywords:** Biomineralization, Porous media, Pore-scale, Microenvironments, Fluid mixing

## Abstract

Microorganisms capable of biomineralization can catalyze mineral precipitation by modifying local physical and chemical conditions. In porous media, such as soil and rock, these microorganisms live and function in highly heterogeneous physical, chemical and ecological microenvironments, with strong local gradients created by both microbial activity and the pore-scale structure of the subsurface. Here, we focus on extracellular bacterial biomineralization, which is sensitive to external heterogeneity, and review the pore-scale processes controlling microbial biomineralization in natural and engineered porous media. We discuss how individual physical, chemical and ecological factors integrate to affect the spatial and temporal control of biomineralization, and how each of these factors contributes to a quantitative understanding of biomineralization in porous media. We find that an improved understanding of microbial behavior in heterogeneous microenvironments would promote understanding of natural systems and output in diverse technological applications, including improved representation and control of fluid mixing from pore to field scales. We suggest a range of directions by which future work can build from existing tools to advance each of these areas to improve understanding and predictability of biomineralization science and technology.

## Introduction

Biomineralization is a widespread process by which organisms produce minerals as part of their metabolism. Here, we focus on extracellular biomineralization, also called biologically induced-mineralization, and its role in shaping biophysical processes in natural and engineered porous media (e.g., soils, aquifers, concrete). The most studied extracellularly formed minerals are silicates, carbonates and metal oxides. Some macroorganisms, such as mollusks, are capable of extracellular biomineralization (Skinner and Jahren, 2005), though the majority is performed by microbes (van Cappellen 2003; Philipps et al., 2013; Dhami et al. 2013; Zhu and Dittrich 2016; Anbu et al. 2016; Gahlawat and Choundhury, 2019). In fact, bacterial biomineralization may have produced the Earth’s oldest macroscopic fossils. Nearly all modern conical stromatolites display a characteristic spacing between cones, consistent with spacing found in ancestral stromatolites formed 2.8 billion years ago (Petroff et al. 2010). This remarkably consistent spacing has been attributed to the periodic rhythms of mineral precipitation, driven by the daily metabolic cycles of photosynthetic cyanobacteria (Petroff et al. 2010). Thus, the spatial organization of biomineralization is dependent on microbial responses to dynamic environmental conditions. In this review, we focus on the dynamics of extracellular biomineralization, which we now refer to simply as "biomineralization", by bacteria in a markedly heterogeneous and spatially structured environment: porous media.

A mechanistic understanding of biomineralization dynamics is not only important for interpreting Earth’s chemical and geological records (Pérez-Huerta et al. 2018), but also for the success of several environmental and industrial applications. Considered an environmentally friendly process, biomineralization has been applied towards the removal of harmful metals and radionuclides from soil (Gavrilescu et al. 2009; Spycher et al. 2011; Li et al. 2016), the extraction of valuable metals from rock and mine waste (i.e., biomining) (Johnson 2014), the removal of ions and hydrocarbons from wastewater and polluted sites (Atekwana and Aal 2015), geological sequestration of CO_2_ (Cunningham et al. 2009; Phillips et al. 2012), enhanced oil recovery (Zhu et al. 2013), and the remediation of building materials such as ornamental stones and concrete (Jiménez-López et al. 2007; De Muynck et al. 2010). A growing number of geomechanical applications have used biomineralization to consolidate and stabilize soil and rock (Mitchell and Santamarina 2005; Ivanov and Chu. 2008; Chou et al. 2011; DeJong et al. 2013; Salifu et al. 2016; Mujah et al. 2017) and to mitigate seismic-induced soil liquefaction (Burbank et al. 2011; Han et al. 2016; Xiao et al. 2018; Zango et al. 2018). Biomineralization has also been considered for large-scale environmental applications such as the sealing of geologic formations produced by fracking (Phillips et al. 2013).

The promise of understanding and harnessing biomineralization at geologically and technologically relevant scales (i.e., mesoscale, meters and kilometers) has motivated interest in the spatial distribution of biomineralization, which is controlled by pore-scale processes. Biomineralization occurs within the pores or cracks of porous or fractured media, which are matrixes of solid grains and void spaces (i.e., the pores or cracks). The structure of these void spaces varies considerably, and their volumes can be occupied by diverse physical and chemical microenvironments. Whether these voids contain a single or multiple fluid and/or gas phases determines whether biomineralization occurs under saturated (i.e., no continuous gas phase) or partially saturated conditions. These differences in hydration state and phase distribution within the porous media dictate an array of other constraints on gas transport and biological function (Tecon and Or 2017). The high degree of heterogeneity in porous media challenges ability to spatially control the extent, rate and uniformity of biomineralization for desired outcomes.

**Fig. 1:**
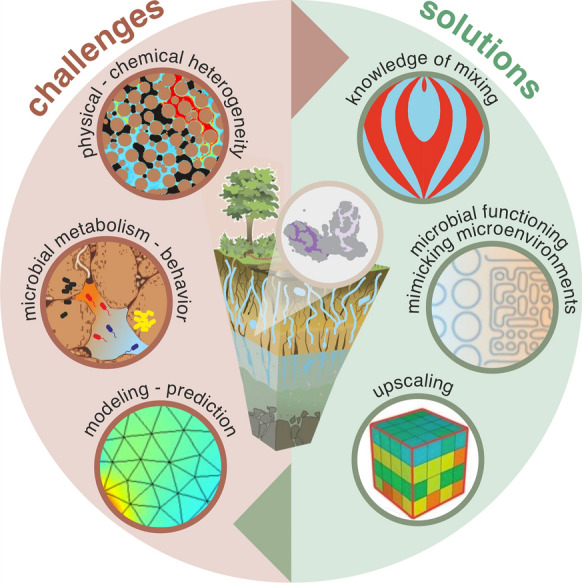
Biomineralization in natural (e.g., soils and aquifers) and engineered (e.g., building materials) porous systems is controlled by multiple interacting components: fluid flow dynamics, environmental chemistry, microbial activity and mineralogy of the solid substrate. The intrinsic heterogeneity of porous media affects all these processes, rendering the prediction and control of extracellular biomineralization processes highly uncertain. We propose several potential solutions requiring further research on the understanding of mixing in the porous media and microbial behavior in heterogeneous microenvironments, and the inclusion of pore-scale process in large scale modeling approaches (upscaling)

The specific objectives of this review are: (i) to disentangle the numerous factors (e.g., fluid flow dynamics, environmental chemistry and microbial ecology) that contribute to microenvironment heterogeneity and currently complicate the predictability of bacterial biomineralization, and (ii) to outline experimental and computational strategies for integrating existing tools and new approaches towards improving spatial control over biomineralization in porous media (Fig. 1). We focus primarily on microbially-induced carbonate precipitation in porous subsurface environments as a model system. However, many other systems are porous (e.g., bones, filters, concrete, cultural heritage monuments) and would benefit from the concepts and insights outlined here.

## Key ingredients for biomineralization in porous and fractured media

The mechanisms that promote or suppress biomineralization in porous media are intimately linked to physical and chemical heterogeneity, fluid flow dynamics and microbial ecology. Here, we focus on pore-scale processes that affect the immediate environment surrounding bacterial cells and discuss how each of these factors contribute to pore-to-pore variability in biomineralization rates, producing challenges for reliable predictions or engineered applications of biomineralization at meso- or field-scale.

### Microbial metabolism

From soils to sediments, aquifers to hot springs, and lakes to oceans, microorganisms produce a large variety of biominerals, including phosphates, silicates, carbonates and oxides, and sulfates and sulfides of various metals (Sarikaya 1999; Riding and Awramik 2000). Biominerals may differ distinctly from their inorganically formed equivalents in shape (Mann 2000), size, crystallinity, isotopic, and trace element composition (Heim 2011). Evidence suggests that biomineralizing microorganisms are ubiquitous in all geologic environments (i.e., soils and rocks) and that their activity plays important roles in the functions of local and global ecosystems (Brussaard 1997). Biominerals are key components of global biogeochemical cycles and serve as critical indicators of past environmental conditions when observed in geological and fossil records (Weiner and Dove 2003).

Microbially-induced carbonate precipitation (MICP) is one of the most studied process of bacterial biomineralization in porous media and the most abundant globally, accounting for up to 42% of the total carbon on Earth (Ehrlich 1998). Carbonate formed through MICP fixes atmospheric CO_2_ and is thus a major sink of this greenhouse gas (Prentice et al. 2001). Microorganisms can catalyze the precipitation of carbonate via different metabolic pathways, including urea hydrolysis, denitrification, ammonification, sulfate reduction, methane oxidation and photosynthesis (see Eq. 1–6) (Achal et al. 2015).

1 $$\begin{gathered} {\text{Urea hydrolysis:}}\quad {\text{ CO}}\left( {{\text{NH}}_{2} } \right)_{2} + {\text{H}}_{2} {\text{O}}{\mathop \rightarrow_{\text{urease}}}{\text{}}2{\text{NH}}_{3} + {\text{CO}}_{2} \\ {\text{NH}}_{3} + {\text{H}}_{2} {\text{O}} \leftrightarrow {\text{NH}}_{4}^{ + } + {\text{OH}}^{ - } \\ {\text{CO}}_{2} + {\text{OH}}^{ - } \leftrightarrow {\text{HCO}}_{3}^{ - } \\ {\text{Ca}}^{2 + } + {\text{HCO}}_{3}^{ - } + {\text{OH}}^{ - } \leftrightarrow {\text{H}}_{2} {\text{O}} + {\text{CaCO}}_{3} \\ \end{gathered}$$

2 $$\begin{gathered} {\text{Denitrification:}} \quad { }1.25{\text{CH}}_{2} {\text{O}} + {\text{NO}}_{3}^{ - } \to 1.25{\text{CO}}_{2} + 0.5{\text{N}}_{2} + 0.75{\text{H}}_{2} {\text{O}} + {\text{OH}}^{ - } \hfill \\ {\text{Ca}}^{2 + } + {\text{CO}}_{2} \left( {{\text{aq}}} \right) + 2{\text{OH}}^{ - } \to {\text{CaCO}}_{3} + {\text{H}}_{2} {\text{O}} \hfill \\ \end{gathered}$$

3 $${\text{Amonification: }} {\text{Amino acids}} + {\text{O}}_{2} + {\text{Ca}}^{2 + } \to {\text{NH}}_{4}^{ + } + {\text{CaCO}}_{3} + {\text{H}}^{ + }$$

4 $${\text{Sulfate reduction: }} 6{\text{CaSO}}_{4} + 4{\text{H}}_{2} {\text{O}} + 6{\text{CO}}_{2} \to 6{\text{CaCO}}_{3} + 4{\text{H}}_{2} {\text{S}} + 2{\text{S}} + 11{\text{O}}_{2}$$

5 $${\text{Methane oxidation: }} {\text{CH}}_{4} + {\text{SO}}_{4}^{2 - } {\text{ Ca}}^{2 + } \to {\text{H}}_{2} {\text{O}} + {\text{CaCO}}_{3} + {\text{H}}_{2} {\text{S}}$$

6 $${\text{Photosynthesis: }} 2{\text{HCO}}_{3}^{ - } + {\text{ Ca}}^{2 + } \to {\text{CH}}_{2} {\text{O}} + {\text{CaCO}}_{3} + {\text{O}}_{2}$$

MICP is performed by diverse microorganisms (bacteria, fungi, algae and metazoans) (Gadd 2010) and contributes to major geological processes, including carbonate sediment formation and lithification and dolomite precipitation (Sánchez-Román et al. 2011; Zhang 2020). This review focuses on bacterial carbonate biomineralization in porous media, though many microorganisms, including fungi and algae (Table 1), catalyze reactions and produce precipitates important to the chemistry and structure of their environments.

**Table 1:** Metabolic pathways of carbonate precipitation in diverse microorganisms

Metabolic pathway	Species	References
Urea hydrolysis	Bacteria	
	*Sporosarcina pasteurii*	Cunningham et al. (2009); Chou et al. (2011); Cuthbert et al. (2012); Ghosh et al. (2019); Mortensen et al. (2011); Martin et al. (2012); Terzis & Laloui (2018); Dawaoud et al. (2014a,b); Zhao et al. (2014); Schultz et al. (2011); Al Qabany et al. (2012); Zambare et al. (2012); Lauchnor et al. (2013); Hommel et al. (2016); DeJong et al. (2014); Gomez et al. (2016); Nassar et al. (2018); Barkouki et al. (2011); Martinez et al. (2014); Ebigbo et al. (2012); Wang et al. (2019); Fridjonsson et al. (2011); Lauchnor et al. (2015); Whiffin et al. (2007)
	*Bacillus cohnii*	Zhang et al. (2017)
	*Bacillus subtilis*	Sarkar et al. (2015)
	*Escherichia coli*	Connolly et al. (2013, 2015)
	*Pseudomonas aeruginosa*	Connolly et al. (2013); Bai et al. (2017)
	*Bacillus sphaericus*	De Muynck et al. (2011, 2013); Cheng et al. (2013); Wang et al. 2012b)
	*Sporosarcina psychrophila*	De Muynck et al. (2013)
	*Terrabacter tumescens*	Li et al. (2016)
	*Bacillus megaterium*	Lian et al (2006)
	Algae	
	*Mychonastes sp.*	Ariyanti & Handayani (2012)
	*Chlorella sp.*	Ariyanti & Handayani (2012)
Ammonification	Bacteria	
	*Myxococcus xanthus*	Jiménez-López et al. (2007); Rodriguez-Navarro et al. (2012); Chekroun et al. (2004)
	*Brevundimonas diminuta*	Rodriguez-Navarro et al. (2012)
Denitrification	Bacteria	
	*Halomonas halodenitrificans*	Martin et al. (2013)
	*Pseudomonas aeruginosa*	Erşan et al. (2015)
	*Diaphorobacter nitroreducens*	Erşan et al. (2015)
	*Pseudomonas stutzeri*	Singh et al. (2015)
	Bacteria	
Sulfate reduction	*Desulfovibrio*	Atlas and Rude (1998)
	Bacteria	
Methane oxidation	Methanosarcinales	Nauhaus et al. (2002)
	*Desulfococcus*	
Photosynthesis	Cyanobacteria	
	*Synechococcus*	Southam (2000)
	*Nostoc punctiforme*	Seiffert et al. (2014)
Unspecified pathway	Bacteria	
	*Arthrobacter sulfonivorans*	Keiner et al. (2015)
	Fungi	
	*Aspergillus nidulans*	Menon et al. (2019)
	*Knufia petricola*	Seiffert et al. (2014)

The rates of bacterial biomineralization are orders of magnitude higher than that of mineral precipitation under abiotic conditions (Zhu and Dittrich 2016; Prasianakis et al. 2017). Typical abiotic rates of CaCO_3_ (calcite) precipitation range from 1 10^−10^ to 6.5 10^−9^ g cm^−2^ s^−1^ for spring-fed streams and from 10^−20^ to 10^−8^ g cm^−2^ s^−1^ for deep sea sediments (Sanjuan and Girard 1996). In contrast, rates of carbonate production by urea hydrolysis range from 3.5 10^–6^ to 1.4 10^−5^ g cm^−2^ s^−1^ with *Bacillus* species (Chu et al. 2012) and from 9.3 10^−9^ to 5.1 10^–8^ g cm^−2^ s^−1^ with *Sporosarcina pasteurii* (Cuthbert et al. 2012). Single-cell biomineralization rates have been estimated to be 7.2 10^−13^ and 16 10^−13^ g Ca^2+^ h^−1^ cell^−1^ with *S. pasteurii* and *Bacillus pasteurii*, respectively (Ganendra et al. 2014).

**Fig. 2:**
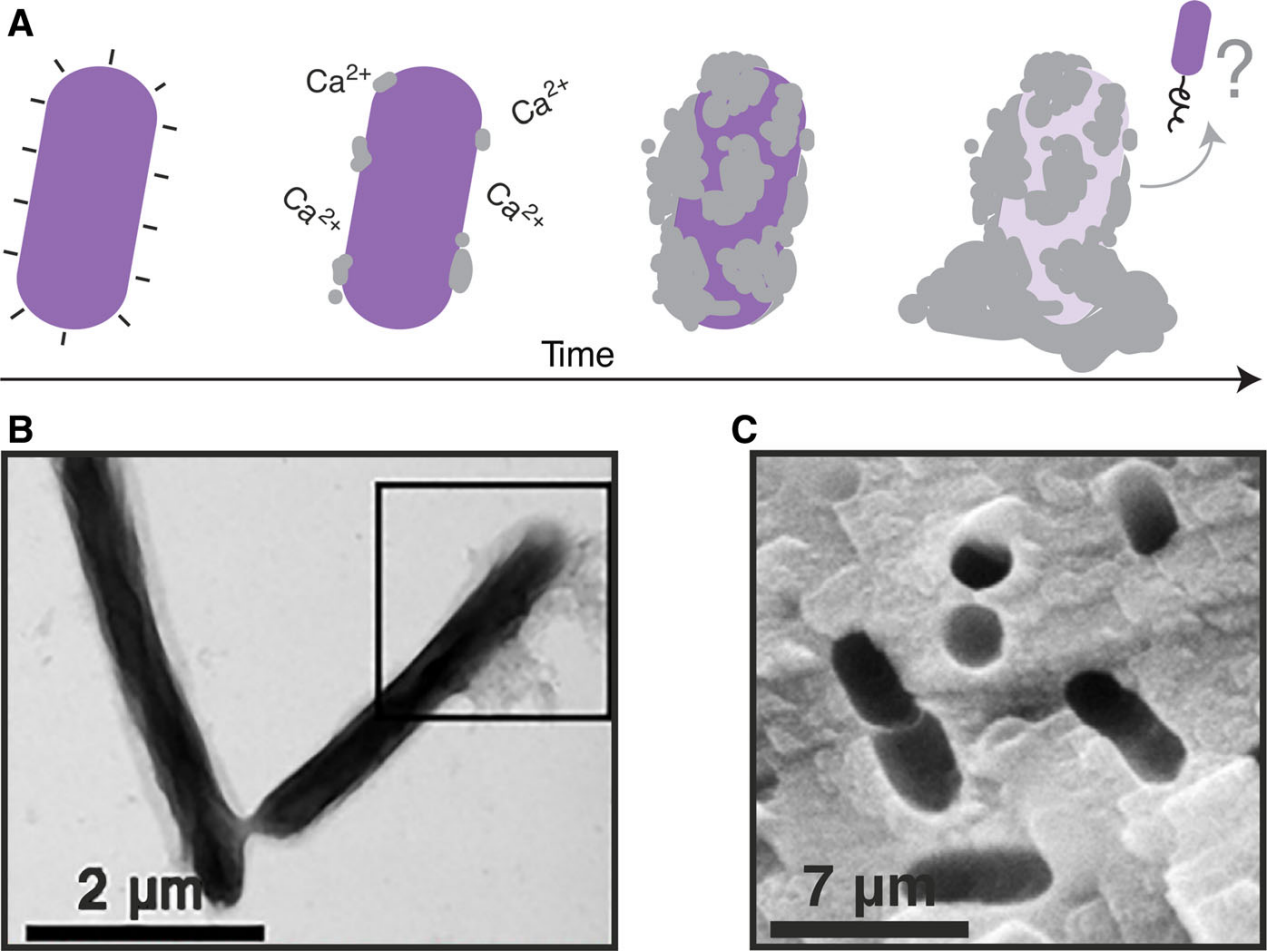
Microbially-induced mineral precipitation. **a** Precipitate, although distributed heterogeneously at the cell wall, encapsulates the bacterium over time. Ca^2+^ ions in the solution are attracted to the bacterial cell wall due its negative charge. The presence of Ca^2+^ ions can result in local supersaturation and precipitation of calcium carbonate on the bacterial cell wall. Imprints of bacteria appear in these minerals, either due to cell death or cell migration after substantial precipitation. **b** Electron microscopy image of *S. pasteurii* cells. The inset square indicates the formation of CaCO_3_ coating the cell surface ( adapted from Ghosh et al. 2019) **c** Bacterial “voids” within the calcium carbonate (adapted from De Muynck et al., 2010)

The rate enhancement achieved by biotic mineralization is facilitated by individual cells and bacterial biofilms, which are aggregates of cells residing within a self-produced extracellular matrix (Conolly et al., 2015; Keren-Paz et al. 2018). Both can catalyze biomineralization by two primary processes. In the first, mineral precipitation is a by-product of bacterial metabolism. For example, bacterial hydrolysis of urea (via urea amidohydrolase) produces bicarbonate and ammonium, increasing environmental pH and therefore calcite precipitation when enough dissolved calcium ions are present (Eq. 1). In the second, bacteria can nucleate mineral precipitation on their cell walls or on the extracellular matrix of biofilms (i.e., extracellular polymeric substances, EPS), which provide a scaffold for biomineralization (Zhu and Dittrich 2016; Flemming et al. 2016; Bao et al. 2018; Han et al. 2019). Increased EPS in the environment increases the consumption of calcium from solution by increasing the number nucleation sites (Bains et al. 2015). Because the exact sites of nucleation are dependent on the charge of cell wall or EPS functional groups (Görgen et al., 2021) (Fig. 2a), the precise biochemical composition of EPS (which varies between bacterial taxa) influences the resulting morphology of mineralized carbonate (Braissant et al. 2003; Ercole et al. 2007). Mineralized carbonate generally precipitates on cell surfaces layer by layer (i.e., successive stratification), implying that bacteria can eventually embed themselves in precipitate (De Muynck et al. 2010; Ghosh et al., 2019) (Fig. 2b). Whether the products of biomineralization are of ecological benefit to these bacteria remains unclear (Dhami et al. 2013). Some authors argue that the organisms construct a precipitated environment to their advantage (Ehrlich 1996; McConnaughey and Whelan 1997), whereas others find the precipitation an incidental by-product of metabolism (Knorre and Krumbein 2000).

In either case, bacteria alter the rates of mineral precipitation by altering the local environment. That the rate of biomineralization reactions is strongly dependent on environmental conditions complicates our ability to accurately predict biomineralization rates in environments like porous media, where microscale spatial heterogeneity in many variables is inherent.

### The physico-chemical environment

The porous environment in which bacteria reside (i.e., the host matrix) creates strong gradients in a number of physico-chemical variables, including temperature, salinity, pH, redox conditions, and substrate composition (Hammes and Verstraete 2002; Mortensen et al. 2011; Han et al. 2019). These variables each modulate all biomineralization processes, from the precipitation of minerals (Liang et al. 2015; Zhu and Dittrich 2016; Bindschedler et al. 2016) to the weathering of parent rock minerals (Gorbushina 2007; Parchert et al. 2012; Seiffert et al. 2014).

Increases in temperature generally increase the rate of MICP (Ferris et al. 2004; Mitchell and Ferris, 2005; Tobler et al. 2011). For example, an increase in ambient temperature from 10 to 20 °C increased *B. pasteurii* calcite precipitation by 6% (from 1.96 10^−10^ to 2.1 10^−10^ g cm^−3^ s^−1^), even at low concentrations of urea substrate (Mitchell and Ferris, 2005). Even higher temperatures within the mesophilic range (20–45 °C) enhance microbial activity, mineral nucleation and growth (Nemati and Voordouw 2003). When incubated on agar plates, *Bacillus sphaericus* was observed to produce carbonate at 13% higher rates at 37 °C than 10 °C (De Muynck et al. 2013).

These relations between biomineralization characteristics and temperature serve only as general guidelines. Very high temperatures (> 50 °C) can kill ureolytic microorganisms (Rebata-Landa 2007) and decrease the size of carbonate crystals, from 15 to 20 µm at 25 °C to 2–5 µm at 50 °C (Cheng et al. 2014). Certain organisms, such as *Sporosarcina psychrophila*, do not produce significant amounts of calcium carbonate under mesophilic conditions (Tobler et al. 2011). Additionally, the urease enzyme can function extracellularly (Dupraz et al. 2009b) even at temperatures higher than the mesophilic range (Bachmeier et al. 2002). Calcite produced from purified urease has been observed to increase by 100% between 22 and 50 °C, from 2.5 10^−8^ to 5 10^−8^ g cm^−3^ s^−1^ (Nemati and Voordouw, 2003). In short, while biomineralization depends on temperature, the precise nature of this dependency can be specific to a given species or application.

The salts dissolved in the aqueous phase and the overall ionic strength of fluid within a porous medium can affect biomineralization rates. High salinity increases carbonate precipitation by ureolysis, depending on the bacterial species (Dupraz et al. 2009a,b; Harkes et al. 2010; Rusu et al. 2011). Salinity-dependent changes in mineral precipitation have been attributed to changes in ionic strength that, in turn, affect bacterial attachment to solid surfaces in the porous medium. Bacterial attachment is generally enhanced with higher salinity and ionic strength due to the decrease in repulsive electrostatic forces between bacteria and solid surfaces (Scholl et al. 1990; Foppen and Schijven 2006). A solution of 9 g L^−1^ of NaCl increased *S. pasteurii* attachment to solids by 30% compared to fresh water (Harkes et al. 2010), indicating stronger association of bacterial cells with surfaces and enhanced retention of bacteria in the porous medium. Biomineralization is controlled not only by the concentration of ions but also by the ion species present. Calcium carbonate nucleation takes longer as the ionic radii of background ions decreases (Burgos-Cara et al. 2017). The presence divalent cations has also been observed to affect calcite wettability. Calcite surfaces with Sr, Ba or Pb are more hydrophobic, while calcite surfaces with Mg are more hydrophilic, which weakens organic compound adsorption and thus controls the growth and shape of mineral precipitate (Andersson et al. 2016).

The pH and redox conditions of the host matrix greatly influence microbial community composition and activity. From a chemical perspective, the formation of hydroxyl ions (OH^−^) as generated with ammonium ions (NH_4_^+^) during urea hydrolysis — induces an alkaline environment (Eq. 1). Higher pH environments enhance carbonate precipitation (De Jong et al., 2010). Thus, the chemical process of biomineralization enhances further precipitation by increasing the local pH immediately surrounding bacterial cells (Rebata-Landa 2007). However, many bacterial species grow optimally at environmental pH values between 7 and 8. As a result, while higher pH environments may chemically favor precipitation, these same environments may limit the abundance of microbes performing it. Furthermore, solution pH may alter the charge of bacterial cell surfaces, which contain zwitterions, and thus alter bacterial attachment and the distribution of cells within the porous medium.

In addition to pH, the redox conditions are important to biomineralization pathways. Methane oxidation by sulfate-reducing bacteria (Eq. 5) produces calcite precipitation under anaerobic conditions only (Cui et al. 2015). In the presence of dissolved metals, sulfate-reducing bacteria can also precipitate metal sulfides (Fortin et al. 1995; Kimber et al. 2020). In soils and aquifers, local redox conditions depend on oxygen diffusion, which is limited by hydration conditions (as oxygen transport is facilitated by the aqueous phase) and by the consumption of oxygen by plant roots and microorganisms. Redox conditions in soil often fluctuate in response to changes in the activity of microbial respiration pathways and the increase or decrease of different respiration products (Kuzyakov and Blagodatskaya 2015).

That said, some metabolic pathways – like urea hydrolysis (Eq. 1) – can produce calcite precipitates under aerobic and anaerobic conditions. Rates of calcite precipitation by *B. sphaericus* and *S. pasteurii* in anaerobic conditions were comparable to those in aerobic conditions, despite the lack of observable bacterial growth in anaerobic conditions (Mitchell et al. 2019). Thus, it appears that calcite precipitation by urea hydrolysis is not significantly affected by the absence of oxygen, at least in this system for the initial 24 h in culture (Mitchell et al. 2019).

Sediments and rocks are composed of diverse and spatially distributed parent minerals, the composition of which greatly influence biomineralization. For example, carbonate production by *Myxococcus xanthus* and *Brevundimonas diminuta* is strongly dependent on the mineralogy of the solid substrate. Placing either species on a calcium substrate stimulated tenfold greater cell density (cells cm^−2^) than on a silicate surface with a commensurate increase in carbonate production (Rodriguez-Navarro et al. 2012). In addition to substrate mineralogy, the presence of environmental contaminants in the host matrix can also alter the size and solubility of biomineralized precipitates. Strontium, for example, decreases carbonate biomineralization by *B. pasteurii* via ureolysis when present in the environment, likely reflecting a strontium-induced decrease in available active sites for nucleation and crystal growth. It is possible the large ionic radius of the Sr ion disrupts the sterics of  the calcite lattice (Mitchell and Ferris, 2005, 2006).

Changes in physico-chemical properties also affect bacterial production of EPS, affecting the amount of available sites for mineral nucleation. Lower temperatures have been shown to alter bacterial growth and metabolism in a manner that increased the availability of precursors for EPS biosynthesis, thereby increasing EPS production (Gorret et al. 2001). Saline environments are favorable for microbial formation of Mg-rich carbonates (Al Disi et al. 2019). Indeed, environmental shifts from low- to high- salinity has been shown to increase the fraction of carboxylic groups on EPS, suggesting that such shifts could increase the Mg-consuming precipitation of dolomite (Diloreto et al. 2021). At low pH, EPS from *Bacillus megaterium* shows a more dense and compact structure due to altered interactions of intermolecular hydrogen bonds (Wang et al. 2012a). Higher EPS and biofilm production by *Lysinibacillus* sp. YS11 (non-ureolytic metabolic pathway) in both aerobic and anaerobic conditions have been observed when calcium is provided in the medium, suggesting that EPS and biofilm formation are altered by MICP (Lee et al. 2017).

### The spatial context: complex and heterogeneous pore networks

The chemical conditions discussed above occur in a host matrix of porous and fractured media with void spaces and fractures containing variable amounts of microbes, liquid and gaseous phases in variable spatial configurations. The spatial structure of the solid-void architecture and the presence of multiple phases fragment microbial aqueous habitats (Or et al. 2007) create a mosaic of fluid flow velocities, punctuated by preferential paths (high velocities) and stagnation zones (low velocities). Convection by fluid flow dominates chemical and particulate transport in certain porous systems, such as aquifers and wetlands. Thus, in determining flow patterns, solute mixing, and chemical dissolution between fluids and surfaces, the spatial heterogeneity of porous media exerts important effects on the activity of microorganisms, including biomineralization. Biochemical mineral precipitation and dissolution are highly sensitive to the distribution of water saturation and the porosity and permeability of the host matrix. For example, natural soils generally decrease in carbon content and oxygen with depth (Ebrahimi and Or 2016), transitioning from oxic to anoxic conditions with distance from the soil surface (Zhang and Furman 2021). A decrease in porosity and permeability or an increase in water saturation limits the diffusion of oxygen, promoting anoxic conditions and therefore biomineralization by anaerobic microbial communities. Additionally, soils often have dynamic hydrologic regimes across dry–wet cycles. These meso- and microscale spatial variations present a major challenge for predictability of biomineralization in natural systems and the success of practical applications at meso- and field-scale, which generally desire uniform distributions of precipitate.

The degree of water saturation, being the fraction of the void space occupied by an aqueous phase, plays an important role in the spatial distribution of biologically precipitated minerals (Terzis and Laloui 2018). This is particularly true in natural examples of unsaturated porous media, in which water is preferentially retained in crevices, small pores and the sites of contact between grains. These regions thus exhibit increased biomineralization compared to regions without water (Cheng and Cord-Rywisch, 2012; Cheng et al. 2013), producing microscale heterogeneity in the distribution of mineral precipitate. Increasing the degree of water saturation increases the connectivity between pores and therefore accessibility of more regions to solutes and microorganisms. Nevertheless, even under fully liquid saturation conditions, homogeneous biomineralization is not guaranteed due to variable porosity within the host matrix.

Biomineralization is generally greater in regions of higher permeability, which have an increased ability to transport fluids (Dawoud et al. 2014a,b). Larger pores, such as macropores (voids larger than 75 µm), have been associated with deeper and broader areas of biomineralized precipitate (De Muynck et al. 2011). Likely, macropores are locations of preferential flow that can deliver more of the necessary components to favor biomineralization (Bundt et al. 2001). The effective porosity of the host matrix, being the connected fraction of void space within the total soil or rock volume, controls not only the accessibility of fluids (and dissolved nutrients and gases) to different spatial locations within the porous medium, but also the accessibility of microorganisms. The pore size distributions of natural soils and rocks can span from nanometers to centimeters, implying that a considerable fraction of the porous medium is inaccessible to microorganisms based on size exclusion (bacteria: 0.2–10 µm; fungal hyphae: 2–50 µm). Knowing the pore size distribution and connectivity of a host matrix can contribute to effective spatial control of biomineralization, by informing the selection of microorganisms for biomineralization applications and estimates of local variation in permeability within the host matrix.

The texture of a soil, being the size distribution of its primary particles or grains, also contributes to the spatial pattern of biomineralization. Studies in homogeneous porous media have found two limitations imposed by soil texture. First, very fine textures, despite having a larger solid specific surface area than coarse textures, hindered carbonate biomineralization due to their very low permeability. Second, coarsely textured soil, which has a high permeability and bacterial accessibility (Zhao et al. 2014), did not observe significant cementation of mineral precipitate when soil grains are very large (Rebata-Landa 2007). In heterogeneous fractured rock, aperture and roughness control permeability and therefore biomineralization rates and spatial patterns. Because fluid flow velocity in fractured media self-organizes into channels that remain stable, the biomineralized precipitate is distributed in the same manner as fluid flow (El Mountassir et al. 2014; Minto et al. 2016).

### The local ecology: temporal and spatial distribution of diverse bacteria

The ecological implications of biomineralization and implications of local ecology on biomineralization remain an open area of research. It is thought that bacteria eventually become encapsulated by the precipitate (periplasmic encrustation), which limits nutrient and oxygen transfer and ultimately results in cell death, leaving bacteria-shaped “voids” within the bulk precipitate (Fig. 2c) (Tazaki et al. 2003; De Muynck et al. 2010; Cuthbert et al. 2013; Miot et al. 2015). However, some recent evidence suggests that cells and biofilms can detach from the mineral substrate during biomineral growth (Li et al. 2015; Bai et al. 2017). An improved understanding of whether cells die or migrate in these circumstances would serve to inform biomineralization models that consider the distribution of cells within a structured host matrix.

The distribution of single cells within porous media is far from uniform and most likely affects the spatial distribution of biomineralization products. Chemotaxis, the ability to sense and move towards a chemical source (Matthäus et al. 2009; Ahmed et al. 2010), enables bacteria to position themselves along gradients within a fully or partially saturated porous matrix (Godány et al. 2017; Creppy et al. 2019; Ebrahimi and Or 2015; Scheidweiler et al. 2020). Chemotactic bacteria can accumulate in a region of high nutrient concentration and then disperse as the nutrient concentration is decreased by diffusion, flow or microbial metabolism. Such ephemeral pulses of bacterial density may determine the locations and rates of biomineralization.

The microorganisms hosted in porous media are subjected to environmental fluctuations both in space and time (Nguyen et al. 2020), affecting both microbial distributions and activity. Like chemotaxis, bacterial gene expression and metabolism can respond strongly to gradients and fluctuations in porous media (Nguyen et al. 2020). Sudden inputs of nutrient can induce soil bacteria to increase nutrient decomposition for hot moments of minutes to hours (Kuzyakov and Blagodatskaya 2015). Fluid flow can also lead to heterogeneity in gene expression by affecting local concentrations of autoinducers, molecules secreted by bacteria to coordinate biofilm formation based on bacterial population density (Kim et al. 2016). In environments with fluid flow, autoinducers accumulate at concentrations highest at the most downstream regions of a bacterial population (Kim et al. 2016). Thus, spatial heterogeneities in the host matrix can alter local biomineralization rates through affecting the spatial expression of traits like biofilm formation, which can influence biomineralization directly or indirectly by altering fluid flow.

**Fig. 3:**
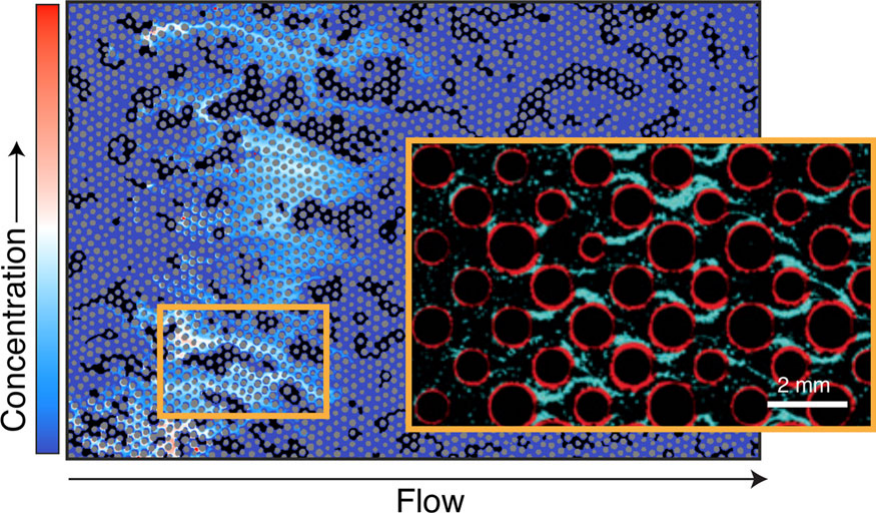
An unsaturated porous media with solid grains (gray), a gas phase (black), liquid phase (dark blue) and chemical concentration gradients. Preferential paths emerge as fluid flow moves around the air pockets. Preferential paths correspond to regions of higher fluid flow velocities and higher chemical concentrations ( adapted from Jiménez-Martínez et al. 2017). Orange inset: The existence of concentration gradients and shear flow control the development of bacterial biofilms. Biofilm differentiation into streamers (cyan) and base biofilm (red) (adapted from Scheidweiler et al. 2019)

Biofilm formation, a prominent trait in many microbial communities, affects the distribution and rates of biomineralization. Biofilms are dense microbial structures, composed of single or mixed species surrounded by a housing of extracellular polymeric substrates (EPS). Functional groups within the EPS have been observed to serve as initial nucleation sites or control the extent of precipitation and the morphology of precipitates (Braissant et al. 2003; Ercole et al. 2007; Decho 2010). Charge density has also been observed for inducing higher nucleation rates (Görgen et al., 2021). Thus, the spatial location of biomineralization in porous media likely depends on the location of biofilms, which are initiated at sites where bacteria attach to surfaces and dependent on chemical gradients (de Anna et al. 2020) and flow (Rusconi et al. 2014; Secchi et al. 2020). Variation in biofilm location and growth in turn contributes further pore-scale heterogeneities in fluid flow and chemistry (Drescher et al. 2013) (Fig. 3). Carbonates formed by biofilms are morphologically distinct from those produced under abiotic conditions (Li et al. 2015), suggesting that biofilm architecture affects precipitate properties. Indeed, carbonate biomineralization appears to form primarily at the base of biofilms (Li et al. 2015). Altogether, biofilms present an excellent example of how the interplay between chemical, physical and biological processes at pore- and single cell-scales produce non-uniform biomineralization at the meso-scale.

## Controlling biomineralization processes in porous media

Efforts to achieve spatial control over biomineralization at useful/application scales have focused on the manipulation of the microbes and/or the pore environment within the host matrix (Antwis et al. 2017). In certain applications, microorganisms capable of biomineralization may already be present in the host matrix (e.g., soil consolidation and stabilization, pollution remediation, ornamental stone consolidation), albeit at low abundance or under unfavorable conditions. In other applications (e.g., geological sequestration of CO_2_, enhanced oil recovery, concrete consolidation), native microorganisms may not be able to biomineralize under prevailing conditions. To overcome these two challenges, applications have sought to stimulate biomineralization: (i) by providing nutrients (biostimulators) to the existing microbial community or chemical amendments designed to select for dominant metabolic activity, (ii) by introducing specific microorganisms to augment native populations (bioaugmentation), or (iii) by a combination of both (Dhami et al. 2017). Together, these methods of enhanced biomineralization have been proposed as environmentally-friendly alternatives to the use of concrete, polymers or resins. Both methods inject solutions of prescribed composition into the host matrix, aiming to induce biomineralization in target regions and circumvent limitations due to uncontrolled bacterial activity or heterogeneous fluid flow and chemical transport. An understanding for how these injected solutions spread and mix with the resident fluids in a host matrix, and particularly how the injected solutions arrive to the microorganisms within the matrix, is key for successful spatial control of enhanced biomineralization strategies.

### Biochemical methods for enhancing specific biomineralization pathways

The primary challenge of enhanced biomineralization is to promote the survival of desired microorganisms in desired locations under often suboptimal host matrix conditions. Current methods have sought to enable specific metabolic pathways or bacterial survivability, through a variety of injected inoculates.

#### Considerations of metabolic pathway

Biomineralization often occurs in extreme chemical and physical environments that stunt bacterial activity and growth. A potential solution for improving activity under suboptimal conditions, is to choose and introduce bacterial species tolerant of specific conditions. For example, desiccation-resistant and aerobic microorganisms, such as ureolytic bacteria and myxobacteria (e.g., *Myxococcus xanthus*), are well suited for near-surface applications, such as the consolidation of ornamental stones and soils (Rodriguez-Navarro et al. 2003; De Muynck et al. 2010; Jonkers and Schlangen 2009). However, oxygen availability limits the long-term use of aerobic microorganisms in deeper parts of geological formations (DeJong et al. 2013), for example, in geological sequestration of CO_2_ or enhanced oil recovery. Controlling the aerated locations is a potential engineering control for promoting an ecological niche for aerobic organisms in an anaerobic subsurface. Currently, injecting air, oxygen, or an oxygenated solution is technically difficult and expensive and remains an area of ongoing work.

Many subsurface applications focus on promoting anaerobic pathways for biomineralization, such as urea hydrolysis and, and more recently, denitrification. Carbonate precipitation by urea hydrolysis (Eq. 1) has been the most studied process for applications and is performed by some facultative anaerobes, such as *B. pasteurii* (Ferris et al. 1997). It is important to note, however, that while urea hydrolysis itself does not require oxygen, some MICP catalyzing organisms may still be sensitive to oxygen availability. Recently, the biomineralization of a prominent MICP model organism, *S. pasteurii*, was found to be inhibited under anoxic conditions (Martin et al. 2012). This finding has led to the exploration of denitrifying bacteria, such as *Halomonas halodenitrificans*, as microbial catalysts for carbonate precipitation (Martin et al. 2013).

#### Bacterial growth and survivability

Uncontrolled microbial growth is a recurrent challenge, as the overgrowth of microorganisms ultimately limits the long-term effectiveness of biomineralization applications. First, rapid overgrowth often leads to the accumulation of detrimental by-products, such as ammonia produced by urea hydrolysis (Eq. 1). Excessive ammonia leads to the eutrophication and acidification of ecosystems, amounting to toxic effects on humans, animals and vegetation. Ammonia can also discolor stone and is thus counterproductive in applications like ornamental stone consolidation (Sutton et al. 2009; Tobler et al. 2011). Second, uncontrolled microbial overgrowth also diminishes the spatial extent of biomineralization. In biostimulation, rapid growth can deplete the biostimulator before it can reach and induce biomineralization at further locations in the host matrix. Improved temporal control over the injection of biostimulator can enhance control over microbial growth by limiting nutrient availability over the course of application (Zhu and Dittrich 2016; Sect. 4.2). To control growth such that mineral precipitation can occur more uniformly over time and space, proposed bioaugmentation strategies include the use of inactive cells, such as lyophilized bacteria or spores, which can be viable for up to 200 years (Schlegel and Zaborosch 1993).

Uncontrolled undergrowth or cell death is another challenge, particularly in bioaugmentation. Microorganisms introduced to soils often decline in abundance or activity shortly after injection (van Veen et al. 1997). Several environmental factors can limit microbial survival and activity, including high pressure, high temperature, saline conditions, competition or predation from native organisms, and the extreme pH conditions often present in applications such as groundwater decontamination and enhanced oil recovery (Okwada and Li, 2010; Phillips et al. 2015). Some mesophilic bacteria, such as *S. pasteurii*, are recommended for biomineralization applications occurring at pressures up to 7.5 MPa (Mitchell et al. 2013). *S. pasteurii* can also tolerate high salinities (Kuhlmann and Bremer 2002; Mortensen et al. 2011) and catalyzes carbonate precipitation in salinities below sea water (35 g L^−1^) (Dupraz et al. 2009a). *S. pasteurii* is also a favorable species for concrete sealing applications, given its tolerance for high alkalinity and high pH (~ 9) conditions (Mobley et al. 1995; Bang et al. 2001). However, pressures higher than 7.5 MPa inhibit *S. pasteurii* DNA replication and protein synthesis, suppressing metabolic functions and growth (Abe et al. 1999). Thus, anaerobic denitrifiers such as *H. halodenitrificans* have been recommended for biomineralization applications when anoxic and high-pressure conditions coincide (Martin et al. 2013). Spores can also survive exposures to high pressure, such as those associated with injections of supercritical CO_2_ during geological sequestration, though chemical additives to the CO_2_ can reduce their viability (Zhang et al. 2006).

#### Composition of injected inoculates and performance assessment

Given the complexity of controlling microbial activity in porous media, in situ biostimulation or bioaugmentation remains experimental (El Fantroussi and Agathos 2005). Here, we highlight recent findings from controlled conditions that exemplify how the composition of an inoculum can be designed to enhance biomineralization. We first introduce the specific application of self-healing (pre-mixed inoculum) and then highlight more general strategies for the composition of injected inoculates.

Self-healing is a special application in that the host matrix, in particular concrete and mortar, can be pre-seeded with biomineralizing microorganisms in effort to achieve more uniform distribution of precipitate (Seifan et al. 2016; Castro-Alonso et al. 2019). To prepare a self-healing material, spores and other microorganisms with low metabolic activities and extremely long lifetimes can be added during the production of the concrete or mortar (Le Metayer-Levrel et al. 1999; Sarkar et al., 2015; Zhang et al. 2017) and contribute to the long-term durability of the building material. Over time, the precipitates produced by these long-lasting microorganisms continue to maintain the structural integrity of the material within which they are embedded.

Two different self-healing techniques have been proposed: one directly introduces only bacteria to the material and the other immobilizes bacteria within “carriers” that are then mixed into the material. These carriers prevent bacterial movement within the host matrix and can be fabricated from a variety of materials. A recent study compared carriers made of silica gel and polyurethane and found that biomineralization by *B. sphaericus* was two-fold higher when carried by silica gel (Wang et al. 2012b). However, cracked mortar specimens containing polyurethane immobilized bacteria regained up to 60% more strength and were up to 10^2^ times less permeable than cracked mortar specimens containing bacteria in silica gel carriers. Experiments with *Bacillus cohnii* have also highlighted the effectiveness of carriers made from volcanic powders (e.g., perlite), completely healing crack widths up to 0.79 mm within 28 days (Zhang et al. 2017). Carriers made from expanded clays could fully heal cracks of smaller widths (0.45 mm).

Bacterial carriers have also been proposed for biomineralization applications requiring injected inoculates. Hydrogel encapsulation of biomineralizing microorganisms has been explored as a means of providing an advantage for introduced bacteria (Wu et al. 2017). For example, a hydrogel can physically protect bacteria from adverse conditions (El Fantroussi and Agathos 2005) or maintain a higher local concentration of nutrient (e.g., urea) around the bacteria to promote precipitation. The injection of a biostimulator metabolically available only to a co-injected bacterial strain can also provide a metabolic niche to the biomineralizer unused by native local microbiota, offering a potential solution for the long-term amendment of the host matrix with a desired biomineralizer (El Fantroussi and Agathos 2005).

Assessing the success of biomineralization applications is complicated in situ, but a variety of methods enable performance assessment in experimental settings. In the lab, assessment of urea hydrolysis can be performed by measuring the decomposition of urea or the production of ammonium, by visually or chemically measuring calcium carbonate, or by strength or waterproof testing the treated host matrix (Wang et al. 2012b; Wu et al. 2017). In the field, the survival of biomineralizers introduced to a soil may be assessed by targeted quantification of the abundance of the introduced bacteria by 16S sequencing (El Fantroussi and Agathos 2005) or by measuring microbial community diversity (Dhami et al. 2017). Whole community monitoring represents an exciting next step in biomineralization applications, as recent work has begun to demonstrate that some biomineralizers (e.g., *S. pasteurii*) may have synergistic interactions with native organisms in the host matrix that increase carbonate precipitation (Dhami et al. 2017).

### Injection methods for controlling fluid flow and chemical transport

Another strategy to spatially control biomineralization focuses on the how the chemical amendments and inoculates are injected, rather than the specific contents. Recent work has introduced temporal and spatial controls over injections, designed to facilitate the transport of reagents to circumvent undesirable patchiness in precipitate formation. The precipitation of surface scabs is a major limitation of shallow applications, such as the treatment of ornamental stone, and arises from the inability of the injected nutrient solution (i.e., urea in growth medium) to penetrate regions farther from the surface (Le Metayer-Levrel et al. 1999). Similarly, a recurrent problem in subsurface applications is the rapid precipitation of mineral around the injection site, plugging adjacent pores and fractures and preventing deeper penetration of the solution and decreasing biomineralization further from the injection site (Schultz et al. 2011). Lower injection rates with lower reactant concentrations in the injected solution have been demonstrated to improve precipitation efficiency and uniformity in biomineralization applications with *S. pasteurii* (Dawoud et al., 2009a, b; Al Qabany et al. 2012; Zambare et al. 2019). Similarly, injecting bacteria before injecting the cementation fluid produced a more homogeneous precipitate distribution than when both were injected simultaneously (Tobler et al. 2012). Still, controlling the local precipitation at the injection site remains an on-going challenge.

Pulsed injections have recently been proposed to reduce precipitation near the injection inlet. Indeed, the intermittent injection of a ureolytic treatment over recurring cycles has reduced the build-up of carbonate around inlets (Lauchnor et al. 2013; Hommel et al. 2016). It is thought that this pulsing maintains bacterial activity over several days while avoiding uncontrolled growth by interrupting longer no-flow periods of low positive mineral saturation index (SI), which determines whether mineral precipitates or stays in solution, with short high-flow periods, which deliver additional growth media and dissolved mineral (increasing SI). This temporal control over microbial activity enables biomineralization to occur further into the host matrix, improving the efficiency and spatial control.

**Fig. 4:**
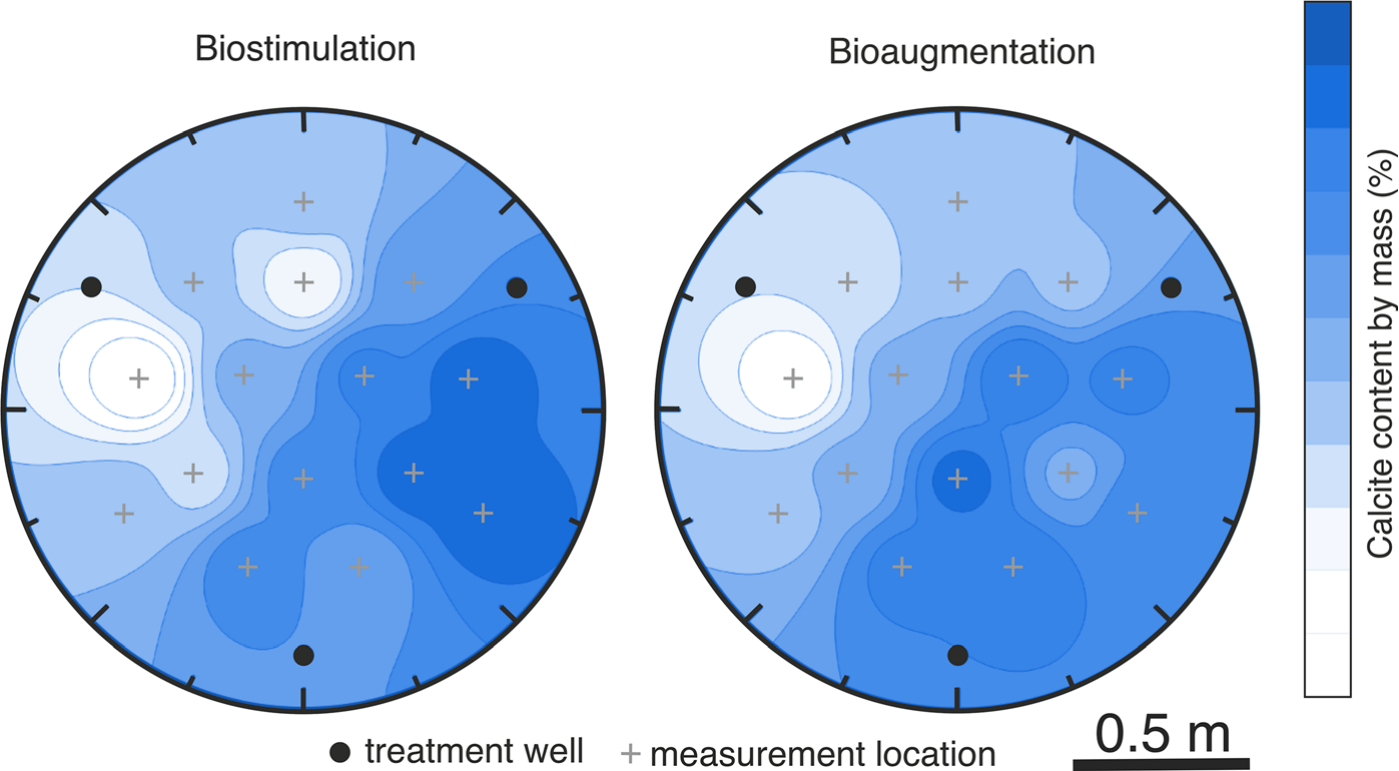
Multi-point injection strategy. Biocementation experiments using a bioaugmentation approach with *S. pasteurii* and a biostimulation approach, which stimulated native ureolytic microorganisms to complete the process. Uniformity in the calcite content was not achieved in any of the approaches. Adapted from Gomez et al. (2016, 2019)

Other injection designs make use of multiple injection inlets, patterning injections to control flow fields and ultimately the location of biomineralization. These multi-point injection designs perform various types of injections: single and multiphase injections, shallow and deep injections, and injections of low and high chemical concentrations (DeJong et al. 2013, 2014; Gomez et al. 2016, 2019; Nassar et al. 2018). By changing which inlets are actively injecting, multi-point injections can change the flow direction within individual pores, creating time-dependent flow fields that promote chemical spreading (Fig. 4). Changing flow fields can also recirculate nutrients around bacteria within the host matrix, shifting conditions towards those of a chemostat. Overall, multi-point injection designs have improved the spatial distribution of biomineralization (DeJong et al. 2014; Minto et al. 2019). Further improvements could be achieved by designing injection programs that produce chaotic mixing (Mays and Neupauer 2012; Neupauer et al. 2014), which would help the field overcome the topologically complex difficulties presented at small scale that currently limit spatial control over biomineralization at meso- and field-scale.

## How modeling tools can guide biomineralization in porous and fractured media

The spatio-temporal heterogeneity in the biological, chemical and hydraulic processes involved in biomineralization make its predictability extremely difficult. While laboratory experiments can provide information about the fundamental mechanisms that control dispersion, mixing and biochemical reactions in porous and fractured media (Kim et al. 2020), they are not able to simultaneously capture all relevant features of natural environments. In particular, individual experiments cannot account for the different types of heterogeneities, i.e., physical, chemical and biological, across different spatial scales (i.e., micrometers to kilometers) (Gelhar et al. 1992). Field-scale transport parameters can differ by orders of magnitude from the values estimated by laboratory experiments, which are by necessity performed on smaller scales (Weber et al. 1992; Vanderborght and Vereecken 2007). The inability to scale experimental results to field applications arises from the hydraulic, geochemical and microbial heterogeneity that exist at each scale and the fact that averaging biomineralization at a single scale (i.e., averaging within a representative elementary volume) cannot fully capture the process at other scales. Thus, the dependency on scale for accurate depictions of these heterogeneities in a porous medium is particularly critical when attempting to predict biomineralization.

To accurately predict biomineralization in heterogeneous porous and fractured media, models need to couple the various processes contributing to biomineralization. A few pore-scale models that consider microscale structure and implement some of the involved processes are found in the literature (Nogues et al. 2013; Qin et al. 2016). However, high computational costs limit the application of pore-scale models to larger scales. In fact, scaling up of biomineralization can be understood as the elimination of pore-scale processes by appropriate averaging of them. At the meso-scale, most of the existing models implement the complex reactions in water as a function of the equilibrium in the chemical system following the mass action law. For example, in urea hydrolysis, the calcite precipitation and dissolution are considered kinetically controlled (Barkouki et al. 2011; Martinez et al. 2014). However, these models do not account for the transport of bacteria and changes in physical or hydraulic properties (porosity and permeability). Models that currently consider changes in physical and hydraulic properties make important simplifications about the kinetic rates that control chemical reactions (Fauriel and Laloui 2012; Cuthbert et al. 2013; Wang and Nackenhorst 220) or assume an immobile and homogeneous distribution of bacteria (van Wijngaarden et al. 2011, 2013, 2016). In some of these works, the simplifications of the kinetics rates are carefully and rationally justified, exemplifying a notable form of upscaling (Fauriel and Laloui 2012). More complex models simultaneously consider multiphase flow, biofilm growth and changing ureolysis rates (Ebigbo et al. 2012; Hommel et al. 2015).

At the field-scale, the scarcity of spatially and temporally distributed information invites the maintenance of simple models, classically based on effective parameters (permeability, dispersivity) (Roden and Scheibe 2005; Cuthbert et al. 2013; Phillips et al. 2016; Cunningham et al. 2019). However, the high degree of heterogeneity and the existence of interfaces induce complex transport and mixing that cannot be captured by this smoothed representation. Fundamentally, these models cannot appropriately account for processes such as mixing and chemical reactions, which intrinsically occur at pore scale (Rolle et al. 2009; Williams et al. 2009; de Anna et al. 2014). Thus, while recent models are starting to couple the processes contributing to biomineralization and some upscaling attempts have been reported (DeJong et al. 2009; Terzis and Laloui 2019), the scaling up of biomineralization still presents a number of challenges to be addressed, such as bacterial attachment (Minto et al. 2019).

## Outlook

Precise control over the spatial distribution of biomineralization in porous media requires holistic consideration of the spatial distribution of physical, chemical and biological factors. Generally, these factors create challenging heterogeneities, that fundamentally shape soils and subsurface ecological processes (Tecon and Or 2017), such as fragmented aqueous phases in unsaturated soils (Or et al. 2007), preferential flow paths that lead to non-uniform transport of nutrients (Le Borgne et al. 2013; Jiménez-Martínez et al. 2015), and highly localized gradients in oxygen and carbon (Borer et al. 2018). Advances in the field should account and systematically control for this complexity.

### Characterization of structure and processes in porous media

Combining recent advances can provide simultaneous quantitative measurements of the various factors and processes that control biomineralization (Robinson et al. 2008). Promising geophysical methods have enabled visualization of dynamic processes such as fluid dynamics and biogeochemical reactions within porous media (Binley et al. 2015). Electrical methods have characterized the physico-chemical environment, such as the spatial distribution of conductive (i.e., iron) (Atekwana and Aal 2015) and non-conductive minerals (i.e., calcite) (Wu et al. 2010). A minimally invasive technique has used spectral induced polarization to monitor the temporal evolution of urea hydrolysis and calcite precipitation in porous media (Zhang et al. 2012). Combining these methods with tracers that report on the metabolic activity of bacteria (Haggerty et al. 2009) are promising avenues by which biochemical reactions and mixing processes may be quantified in porous and fractured environments.

### Microbial activity in heterogeneous porous media

An ongoing challenge is to understand how the spatial and temporal heterogeneity in porous media affect bacterial distribution and function. Pore-scale experiments and simulations have shown that bacterial growth varies considerably in space in the presence of chemical gradients (Knuston et al., 2005). We propose that future attention to how porous media affects motility range and bacterial migration and/or transport will inform efforts to control the spatial distribution of bacterial biomass and therefore biomineralization.

Attention should also be paid to the dynamics of bacterial biomineralization in the presence dynamic local environments. Minute-scale fluctuations in nutrient concentration have been experimentally shown to induce fluctuations in the growth rate of *E. coli*, leading to differences in net growth compared to steady environments of equal average nutrient availability (Nguyen et al. 2021). Similar fluctuations in porous media may change biomineralization rates over time (e.g., through changes in growth rate). It is also possible that diverse soil microorganisms may be less responsive to fluctuations. Communities in coastal sediments appear to have evolved the capacity to continuously denitrify, even as oxygen (generally a denitrification inhibitor) fluctuates around them (Marchant et al. 2017). Similarly, some soil communities have been found to grow faster (as seen by higher RNA to DNA ratios) when exposed to fluctuating conditions (oxic/anoxic) than to steady ones (steadily oxic or anoxic) (DeAngelis et al. 2010). How communities can maintain steady activity and growth under fluctuations may provide solutions for biomineralization applications that desire steady precipitation under difficult to control conditions.

### Genetic engineering or experimental evolution of microorganisms

To achieve uniform biomineralization across meters or kilometers, a possible strategy could include the engineering of an organism that regulates biomineralization in a cell density-dependent manner. Bacterial quorum sensing systems, which mediate density-dependent gene expression, have already been manipulated to improve the treatment of wastewater and energy production from microbial fuel cells (Yong et al. 2015). Manipulating microbial activity such that higher cell densities, such as those occurring at injection inlets or in biofilms, coincide with lower single-cell biomineralization rates could help prevent uneven formation of precipitates, such as clogging at inlets.

Another possible avenue for spatial control could be the bioengineering of bacterial strains with enhanced dispersal capabilities, such as reduced attachment to surfaces and slower rates of surface-attached colony growth. Reducing the number of cells within a surface-attached colony has been shown to increase the spatial range at which the human pathogen *Pseudomonas aeruginosa* colonizes its host (Laventie et al. 2018). Engineering biomineralizers to disperse more effectively through increased swimming speeds or tumbling rates could similarly spread the distribution of biomineralization throughout a host matrix. Strains of *E. coli* with enhanced motility have been evolved in soft agar environments that facilitate the selection of mutants that disperse the fastest (Ni et al. 2017).

Strains that are more genetically tractable can be metabolically engineered to catalyze specific biochemical reactions. *P. aeruginosa* MJK1 and *E. coli* MJK2 have previously been engineered to perform urea hydrolysis (Connolly et al. 2013). While respectively 4- and tenfold lower than the endogenous ureolytic activities of *S. pasteurii*, the engineered strains exhibited substantial ureolysis rate under standard laboratory growth conditions, but were able to grow 1.5- and twofold faster and to higher population densities. Genetically engineered spore-forming bacteria, such as the alkaliphilic *B. subtilis*, have been developed for self-healing applications (Sarkar et al., 2015). For applications of specific pH, candidate organisms include mutants of the fungus *Aspergillus nidulans* MAD1445 that can grow and promote calcium carbonate precipitation (Menon et al. 2019). For high temperature and hypersaline applications, strains isolated from hot springs and growing in highly saline environments offer initial candidates for engineered strains that tolerate and biomineralize in such conditions (Fouke 2011; Okumura et al. 2013).

### Abstracting and mimicking natural microenvironments

For pore-scale studies, microfluidics represents a powerful tool to study biomineralization processes at the microscale. Microfluidics offers the ability to precisely control fluid flow and mimic natural microenvironmental conditions, while allowing optical observation and quantification (Schultz et al. 2011; Lauchnor et al. 2013; Yin et al. 2009; Singh et al. 2015). Classic microfluidic materials, such as glass and silicone (Rusconi et al., 2014; Son et al. 2015), may not contain many physico-chemical properties occurring in natural or engineered porous media (Aleklett et al. 2017). The inclusion of a mineral surface would offer the ability to perform microfluidic experiments with substrates that directly reproduce more natural conditions of asperity, wettability, porosity, and heterogeneity. So far, only a handful of devices have been developed to study fluid–solid reactions and mineral leaching (Satoh et al. 2007; Song et al. 2014; Ciceri and Allanore 2015; Osselin et al. 2016; Neuvulle et al., 2017; Jiménez-Martínez et al. 2020), or fluid dynamics (Porter et al. 2015; Singh et al. 2017). Adopting mineral microfluidics for biomineralization studies would enable experiments that include all fundamental characteristics that affect fluid flow, chemical reactions and microbial interactions at the fluid–solid interface.

### Achieving spatially controlled biomineralization at the field scale

**Fig. 5:**
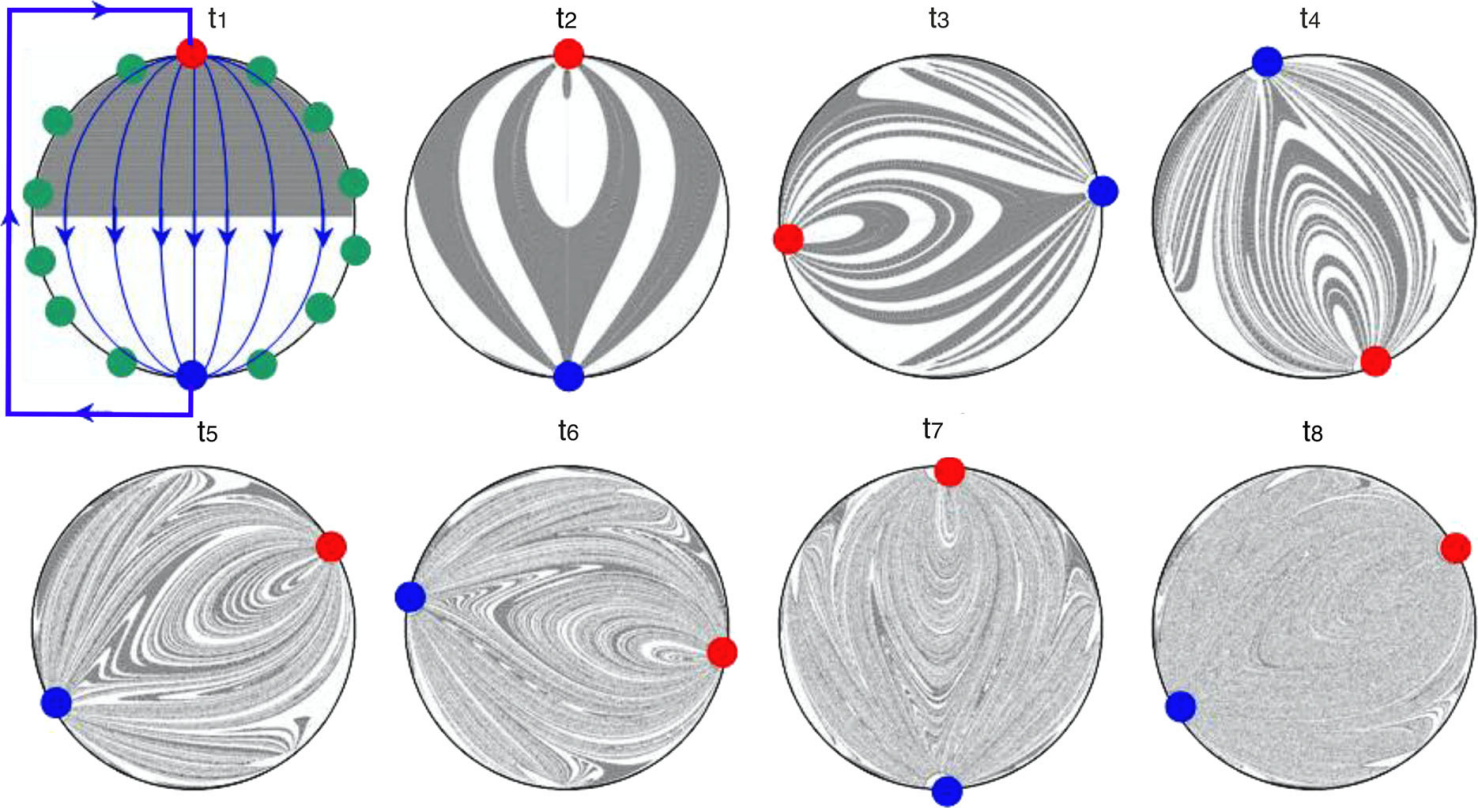
Chaotic mixing in porous media spatially distributes a solution of nutrient and bacteria over time in an optimal stirring protocol. Flow from an injection well (red) to an extraction well (blue). If the time scale of biomineralization (*i.e.*, kinetic) is larger than the time needed to reach the well mixed conditions (t_8_), a more homogeneous spatial distribution of precipitated could be obtained. Adapted from Lester et al. (2009)

Mixing is a combination of stirring, which increases the interfacial area between the resident and the injected solution and creates concentration gradients, and diffusion, which smooths out the concentration gradients and homogenizes the concentration field. Stirring, in particular, can be controlled through designing fluid injection strategies to stimulate biomineralization in a spatially-controlled manner. Multi-point injection strategies can be used to stretch and fold the injected chemical plumes and further spread the inoculum into the host matrix (Mays and Neupauer 2012; Neupauer et al. 2014). Current multi-point injection applications tend to create encapsulating flows, which can isolate fluid zones for lengthy periods (Tefrey et al., 2012). However, mixing can be accelerated by designing injection programs that produce chaotic flows (Lester et al. 2016) (Fig. 5). The design of a multi-point injection program that mixes even in laminar flow conditions represents an immediate challenge that, if solved, can greatly improve our spatial control over biomineralization.

The tendency of bacteria to aggregate or attach near the inlet of injection sites remains a challenge, as it produces spatial heterogeneities in biomineralization applications. Sonication, the use of ultrasounds, have been demonstrated to efficiently produce bacterial suspensions without aggregates (Sanz et al. 2003) and without killing bacteria (Piyasena et al. 2003). Sonication prevents microbial biofilms (Wang et al. 2017) and can increase the rate of bacterial cell growth (Pitt and Ross, 2003). Ultrasonication is already used in soil and sediment remediation (Radu et al. 2020) and could serve as an environmentally friendly (no toxic chemicals are used or produced), low cost, and compact (allowing on-site treatment) solution to improve the spreading of inoculated bacteria across the host matrix (Pham et al. 2009).

Enhanced mixing in unsaturated porous media can be achieved by manipulating the degree of water saturation, which exerts strong control over solute mixing and chemical reactions (Jiménez-Martínez et al. 2015, 2017). When an injected suspension of bacteria and nutrients does not significantly change the degree of saturation, then the bacteria and nutrients in the injection travel through preferential paths in the host matrix. This produces a fingering pattern, and the bacteria and nutrients are then unable to reach isolated clusters of water, resulting in patchy biomineralization due to incomplete mixing. If the water saturation of the porous host matrix is increased before or during injection, the accessibility of the porous media to bacteria and nutrient increases.

Some environments cannot be saturated. To enhance mixing in unsaturated environments, we propose two possibilities: (i) a simultaneous injection of an immiscible phase (e.g., air) to enhance mixing of the injected inoculum with the resident fluid (Jiménez-Martínez et al. 2016); and (ii) a forced desaturation (e.g., by evaporation) of an initially low concentration solution to concentrate the inoculum into several small water volumes (McLean et al. 1997). The latter proposition would still produce patchy mineralization, but in smaller patches that are more homogeneously distributed as controlled by the texture of the host matrix.

### Harnessing the predictive power of numerical tools

From a modelling perspective, the key challenge is the inclusion of the effects of pore-scale processes and bacterial behavior into multi-scale numerical models.  The appropriate averaging of these small-scale processes would allow upscaling by eliminating the need to model them explicitly. Advection–dispersion model is commonly used in continuum models to simulate the transport of bacteria through porous media, and more recently, chemotaxis has been incorporated as an additional advection-like term (Adadevoh et al. 2017). However, this approach fails to predict the bacterial residence time and distribution in the host matrix, and therefore the rate of biologically-driven reactions. Furthermore, models typically employ simplified kinetics that do not account for cell density, pH effects or product inhibition. Thus, the reaction rate and therefore the mass of precipitate produced under natural conditions differs by orders of magnitude with respect to rates calculated under well-controlled laboratory conditions (i.e., batch experiments from which kinetics are measured). Because the rate of biomineralization changes in space and time, it is very difficult to know *a priori*. This fact reduces the predictive capacity of current numerical models. Recent advances in multispecies reactions modeling developed for geochemical purposes (Valdes-Abellan et al. 2017), along with new theories coupling complex fluid dynamics with transport processes in both fully and partially saturated porous media (e.g., lamella-based model) (Le Borgne et al. 2013, 2014, 2015; Jiménez-Martínez et al. 2017), inform about the mixing of nutrients and chemical amendments and provide a new opportunity to study and predict chemical heterogeneity at meso-scale. The models developed in the last decade for the transport of microorganisms (Creppy et al. 2019) and the growth of biofilms (Ezeuko et al. 2011) in porous media, as well as the biologically induced reactions and clogging processes (Thullner et al. 2002; Brovelli et al. 2009) will serve as the basis to complement and optimize the meso-scale models of biomineralization.

## Summary

Biomineralization processes have been intertwined with the origins of life on Earth, as evident in the geologic record. An improved understanding of biomineralization processes in porous media requires a pore-scale integration of the physical and chemical micro-environments that contribute to its spatial heterogeneity. An improved understanding of how the spatial heterogeneity of porous media affects biomineralization would improve our interpretation of ancient and ongoing natural processes and promises for improved control over several technological applications that rely on biomineralization. Immediate challenges towards this perspective include an improved understanding of microbial behavior in heterogeneous microenvironments, using a pore-scale understand to inform the control of fluid mixing at field-scale, and the upscaling of microscale processes in predictive tools. By integrating these elements, we can then develop a predictive understanding of biomineralization in porous media, its rate and spatial distribution, in nature and in practice.
